# Evaluation and Experiment of High-Strength Temperature- and Salt-Resistant Gel System

**DOI:** 10.3390/gels11080669

**Published:** 2025-08-21

**Authors:** Changhua Yang, Di Xiao, Jun Wang, Tuo Liang

**Affiliations:** 1 School of Petroleum Engineering, Xi’an Shiyou University, Xi’an 710065, China; ych569@126.com (C.Y.); xd1181163575@163.com (D.X.); 2 Engineering Research Center for Development and Control of Low-Permeability and Ultra-Low-Permeability Reservoirs in Western China, Ministry of Education, Xi’an 710065, China; 3 China National Petroleum Chang Qing Oilfield Company’s New Energy Division, Xi’an 710016, China; wangjun21_cq@petrochina.com.cn

**Keywords:** response surface method, thermogravimetric analysis, microscopic visualization, nuclear magnetic analysis, parallel displacement

## Abstract

To address the issues of poor thermal stability, inadequate salt tolerance, and environmental risks in conventional gel systems for the development of high-temperature, high-salinity heterogeneous reservoirs, a triple-synergy gel system comprising anionic polyacrylamide (APAM), polyethyleneimine (PEI), and phenolic resin (SMP) was developed in this study. The optimal synthesis parameters—APAM of 180 mg/L, PEI:SMP = 3:1, salinity of 150,000 ppm, and temperature of 110 °C—were determined via response surface methodology, and a time–viscosity model was established. Compared with existing binary systems, the proposed gel exhibited a mass retention rate of 93.48% at 110 °C, a uniform porous structure (pore size of 2–8 μm), and structural stability under high salinity (150,000 ppm). Nuclear magnetic resonance displacement tests showed that the utilization efficiency of crude oil in 0.1–1 μm micropores increased to 21.32%. Parallel dual-core flooding experiments further confirmed the selective plugging capability in heterogeneous systems with a permeability contrast of 10:1: The high-permeability layer (500 mD) achieved a plugging rate of 98.7%, while the recovery factor of the low-permeability layer increased by 13.6%. This gel system provides a green and efficient profile control solution for deep, high-temperature, high-salinity reservoirs.

## 1. Introduction

With global oil and gas exploration extending into deeper and more geologically complex formations, high-temperature, high-salinity heterogeneous reservoirs have become a critical frontier for enhancing reserves and production [1]. However, their development is hindered by two fundamental challenges. First, elevated formation temperatures and salinities cause severe molecular chain degradation and ion-screening effects in conventional polymer gels, leading to a rapid loss of mechanical strength, dehydration, and shrinkage [2]. Second, pronounced heterogeneity—often with permeability contrasts exceeding 10—results in preferential flow through high-permeability channels [3], leaving low-permeability zones with oil recovery factors below 40%. As a result, the recovery factor of such reservoirs is typically 15–30% lower than that of homogeneous reservoirs, with annual production losses exceeding ten million tons [4]. Existing Cr/aldehyde crosslinked systems offer a degree of thermal stability, but their application is constrained by heavy-metal contamination risks and aldehyde toxicity, highlighting the urgent need for environmentally friendly, intelligent profile-control materials [5]. In recent years, organic–inorganic hybrid gels have attracted increasing attention due to their structural tunability, yet achieving long-term stability under extreme reservoir conditions remains a critical bottleneck, underscoring the necessity for innovative, sustainable solutions [6].

**Figure 1:**
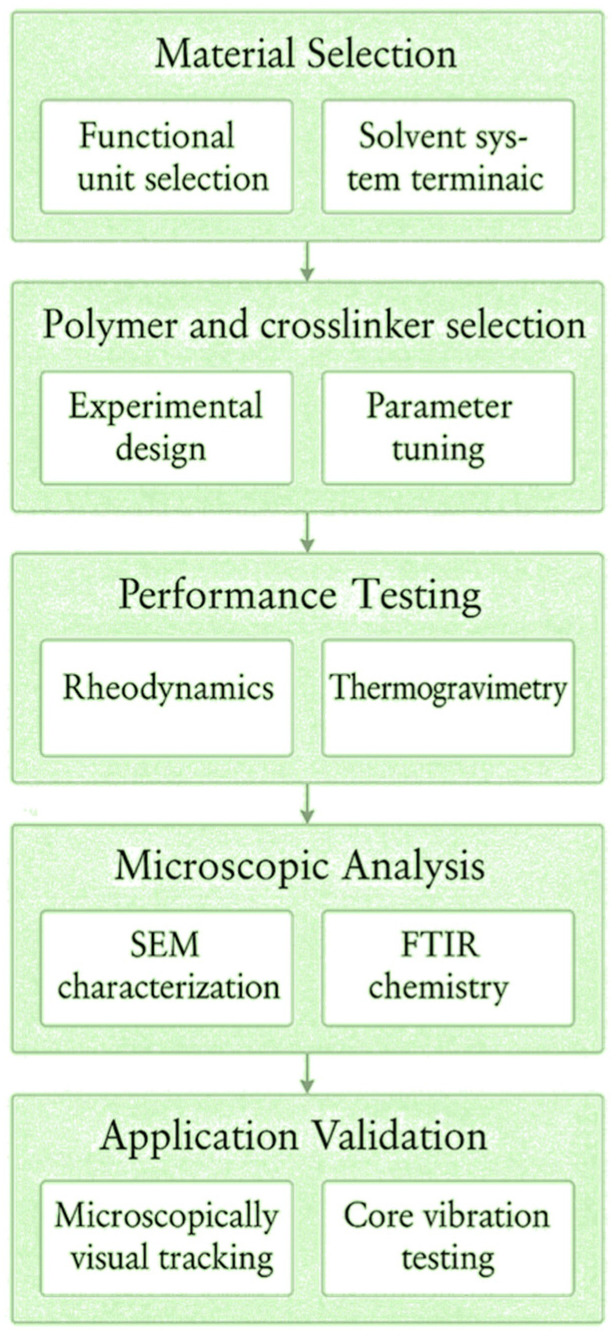
Research roadmap.

For profile control and water shutoff in high-temperature, high-salinity reservoirs, extensive research has focused on composite systems of polyacrylamide (APAM) with organic amine crosslinkers such as polyethyleneimine (PEI) [7]. Anionic polyacrylamide (APAM), owing to its carboxyl ionization capability, is a preferred backbone for salt resistance; however, purely electrostatically crosslinked networks tend to dissociate at elevated temperatures [8]. Polyethyleneimine (PEI), as a polyamine crosslinker, can enhance salt tolerance via charge shielding, yet its thermal stability is limited [9]. Phenolic resin (SMP) can improve the thermomechanical strength of gels but often struggles to reconcile solubility with environmental compatibility [10]. Some studies have introduced a third component into APAM/PEI systems—such as hydrophobic monomers, nanoparticles, or phenolic covalent crosslinkers—to reinforce network stability or improve mechanical performance under salt-screening conditions [11]. However, these modifications typically focus on optimizing a single performance parameter and lack a systematic, multiscale synergy design of crosslinking modes at the molecular, microscopic, and macroscopic levels. Fundamentally, a single crosslinking mechanism is insufficient to break the “strength–stability–environmental compatibility” trade-off [12,13]. To address this, the present study introduces phenolic resin (SMP) with aromatic ring structures into the established APAM/PEI system [14]. During the condensation reaction, SMP forms a covalent backbone with high thermal resistance, while the aromatic hydrophobic domains and π–π interactions create localized hydrophobic microenvironments that reduce water activity and mitigate salt-screening effects [15,16,17,18]. These features, combined with ionic coordination, give rise to a multimodal synergistic network. The novelty of this design lies in a hierarchical construction of crosslinking modes across molecular, microscopic, and macroscopic scales, enabling simultaneous optimization of network stability, toughness, and gelation controllability under harsh high-temperature and high-salinity conditions—rather than a mere additive combination of three components [19,20,21,22,23]. Constructing such a multi-mechanism synergistic molecular network to achieve structurally adaptive regulation under extreme reservoir environments represents a key scientific pathway to overcoming this challenge [24,25]. To validate this strategy, we first optimized the APAM/PEI/SMP composition and preparation conditions. Gel performance was then systematically characterized through rheological measurements, thermal and salt resistance tests, and self-healing/toughness evaluations. The multimodal crosslinking mechanism was elucidated using Fourier transform infrared spectroscopy (FTIR) and scanning electron microscopy (SEM). Finally, the application potential was verified via microscopic visualization, nuclear magnetic resonance (NMR) testing, core-plugging experiments, and oil recovery enhancement evaluations. The overall research workflow is illustrated in Figure 1.

## 2. Results and Discussion

### 2.1. Analysis and Evaluation of Gel System

#### 2.1.1. Two-Factor Interaction in the Response Surface

According to the designed experiments, 30 different combinations were carried out, including 16 factorial points, 8 axial points and 6 center points. Corresponding to the designed center points, 6 groups of experiments were repeated to ensure the validity of the model and reduce the estimated variance, the variance table is shown in Table 1 below. Finally, the quadratic regression polynomial model of Equation (1) was obtained.

G = 92.00 + 1.67A + 0.5833B + 0.25C − 1.333D − 0.5AB − 0.75AC − 1.5AD + 0.5BC + 0.25BD + 0.25CD − 12.17A^2^ − 4.27B^2^ − 4.92C^2^ − 3.54D^2^ (1)

It can be known from the analysis of variance table that the F of the regression model is 9.13 and *p* < 0.0001, indicating that the established regression model is extremely significant and suggesting that the fitting degree of the established quadratic multivariate regression equation is good. The misfitting term F = 6.55 and *p* = 0.0855 > 0.05, indicating that the misfitting term is not significant and proving that there are no misfitting factors in the regression model. Through the significance test of each coefficient in the formula, it can be obtained that the ranking of the influence degrees of the four factors on the gelation strength is as follows: Anionic polyacrylamide (A) > High salt resistance crosslinking agent (B) > Mineralization degree (C) > Temperature (D).

**Figure 2:**
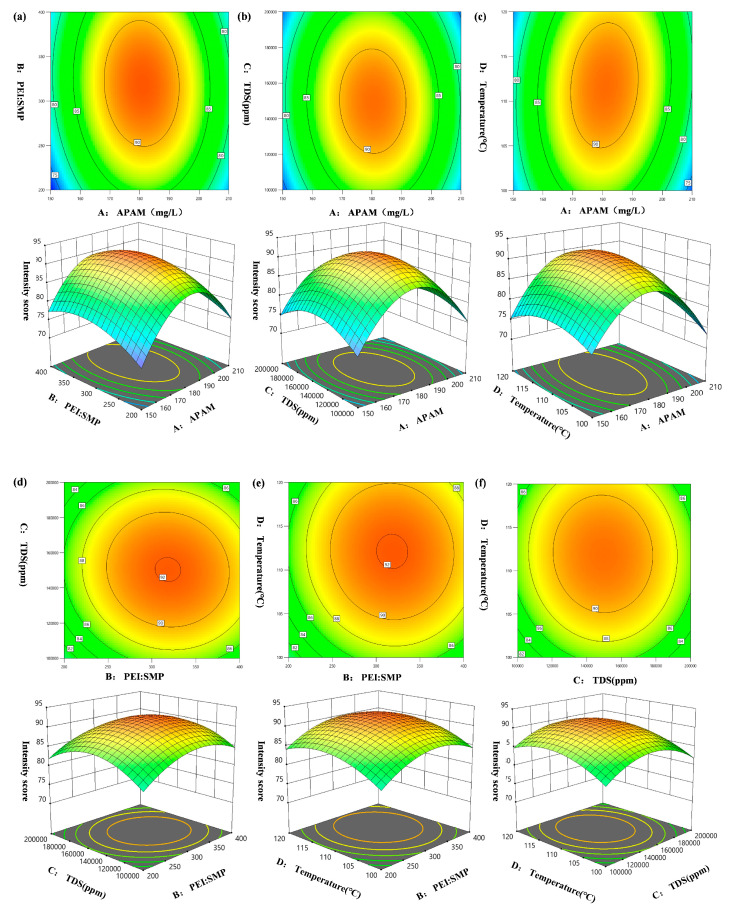
Response surface plot of the two-factor interaction. (**a**) The interaction between factors A and B. (**b**) The interaction between factors A and C. (**c**) The interaction between factors A and D. (**d**) The interaction between factors B and C. (**e**) The interaction between factors B and D. (**f**) The interaction between factors C and D. (The red dots in the figure represent the extreme points).

Figure 2a illustrates the effects of APAM concentration and the PEI:SMP ratio on the strength score of the gel system. In the response surface plot, the degree of curvature reflects the magnitude of influence exerted by the studied factors on the response: The more pronounced the curvature, the greater the effect of APAM concentration on gel strength. The elliptical shape of the contour lines indicates that both APAM concentration and the PEI:SMP ratio are critical factors for achieving high gel strength, with a significant interaction between them; in contrast, circular contours would suggest a less pronounced interaction. The 3D response surface opens downward, showing marked variations with changes in APAM concentration and PEI:SMP ratio, and exhibits a distinct maximum response value, confirming a strong interaction between these two parameters in influencing gel strength. When the amount of APAM is held constant, APAM concentration exerts a unidirectional effect on yield; however, when PEI:SMP is fixed, the effect of APAM concentration on strength becomes more pronounced and bidirectional. Under these conditions, the optimal APAM concentration was determined to be 180 mg/L. To validate the model’s prediction, three independent replicate experiments were conducted. The results showed a gel strength score of 93.5 ± 2.3, in close agreement with the predicted value of 92.0, thereby confirming the model’s reliability and predictive accuracy. As shown in Figure 2b,c, at a salinity of 150,000 ppm and a temperature of 110 °C, the interaction between salinity and APAM concentration is most pronounced. Figure 2d further reveals that the most significant interaction occurs when the PEI:SMP ratio is 3:1. Additionally, it demonstrates the influence of salinity on both APAM concentration and the PEI:SMP ratio. When the temperature is below 100 °C, poor solubility of the reactants leads to incomplete reactions and suboptimal yield. Conversely, at temperatures above 130 °C, excessive solubility causes adverse effects that outweigh the benefits, thereby compromising the stability of gel strength under otherwise optimal conditions.

Figure 2e,f present the interaction effects of PEI:SMP ratio with temperature and of salinity with temperature, respectively. When the temperature is held constant, a PEI:SMP ratio below 3:1 results in incomplete reactions and precipitation of the reactants. At temperatures above 130 °C, excessive solubility of the products leads to a marked decline in gel strength. In contrast, the interaction between salinity and temperature during the reaction process is not significant, consistent with the variance analysis results shown in Equation (1). Based on these findings, the optimal reaction temperature was determined to be 110 °C.

#### 2.1.2. Time–Viscosity Relationship Fitting

**Figure 3:**
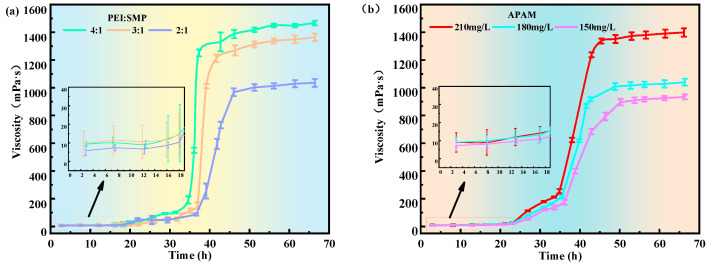
Viscosity–time Boltzmann fitting diagram under different main control factors. (**a**) A1: 180 mg/L APAM. (**b**) A2: PEI:SMP = 3:1.

As described in Section 2.1.1, to establish a direct correlation between crosslinker concentration and both system viscosity and gelation time, the time–viscosity data corresponding to different crosslinker concentrations were subjected to nonlinear surface fitting using GAUSS, COSINE, and other fitting functions. Figure 3 below shows the fitted relationship between time and viscosity.

The fitted functions yielded coefficients of determination (R^2^) ≥ 0.8, indicating that the regression deviations provided a reliable explanation of the overall variance and that the model effectively captured the general trend of viscosity evolution. Under different formulation conditions, the variations of $t_{0}$ and ∆t were closely related to APAM concentration and the PEI:SMP ratio: Increasing APAM concentration markedly shortened $t_{0}$, thereby accelerating gelation, whereas increasing the PEI:SMP ratio enhanced $\eta_{max}$, improving the final gel strength. A key advantage of this fitted equation is that it allows the extraction of critical parameters from a limited number of short-term experiments, enabling the prediction of gelation states under various shut-in times and thus providing a scientific basis for field operations. For example, by adjusting the APAM concentration and crosslinker ratio, $t_{0}$ can be controlled within the permissible shut-in duration on site, ensuring that the gel fully forms in the target zone without premature failure.

### 2.2. Test and Characterization of Gel System

#### 2.2.1. SEM Electron Microscope Image Analysis

**Figure 4:**
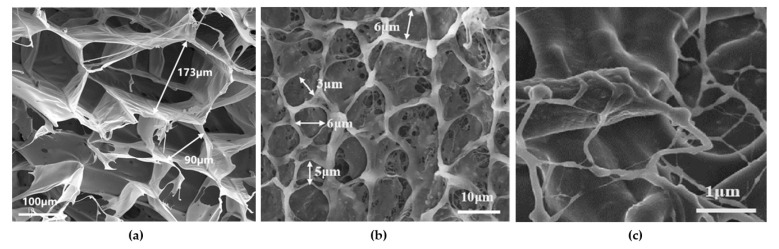
Scanning electron microscope images of gels of different sizes. (**a**) 100 μm. (**b**) 10 μm. (**c**) 1 μm.

After the gel system is formed under static conditions, it exhibits an irregular and complex three-dimensional network structure, as shown in Figure 4. The formation of this structure primarily arises from the interactions between the carboxyl groups of the APAM polymer chains and the protonated amine groups of the PEI crosslinker. Through a combination of crosslinking and molecular entanglement, these interactions construct a compact and interconnected network.

Microscopic observations at different magnifications revealed that the three-dimensional network contained pores ranging from 2 μm to 8 μm in diameter. These pores impart a distinct porous morphology at the microscale, forming a complex structure capable of both encapsulating free water and binding bound water. Such structural characteristics substantially enhance the overall performance of the gel, endowing it with excellent water absorption and retention capabilities while also improving volumetric stability. To adapt the gel for high-temperature, high-salinity conditions, a structural modification strategy was implemented. The amide groups (–CONH_2_) of APAM and the primary amine groups (–NH_2_) of PEI undergo covalent condensation with the hydroxymethyl groups (–CH_2_OH) of the high-temperature stabilizer SMP, thereby forming a thermally stable backbone. This approach minimizes premature chain scission and degradation of the viscous polymer solution during gelation at elevated temperatures. Concurrently, the incorporation of SMP further increases the compactness of the gel structure. The SMP molecules, with their aromatic rings, promote strong interchain entanglement and association, resulting in tighter intermolecular interactions. As a consequence, the entire gel network exhibits higher structural stability under external stress, forming a stereoscopic lattice with SMP aromatic rings as nodal hubs. This vividly illustrates the synergistic mechanism of “electrostatic salt resistance, covalent thermal stability, and aromatic ring water locking.” Even under applied mechanical forces, the three-dimensional network accommodates a certain degree of deformation due to its inherent compressible void space. This property acts as a buffering mechanism, effectively dissipating the applied stress and minimizing structural damage caused by excessive strain. Furthermore, the reduced pore spacing increases molecular shielding in space and strengthens the interlocking of the network, ensuring that, after gelation, the overall structure retains high stability while exhibiting enhanced mechanical strength.

#### 2.2.2. Infrared Spectroscopy Analysis

**Figure 5:**
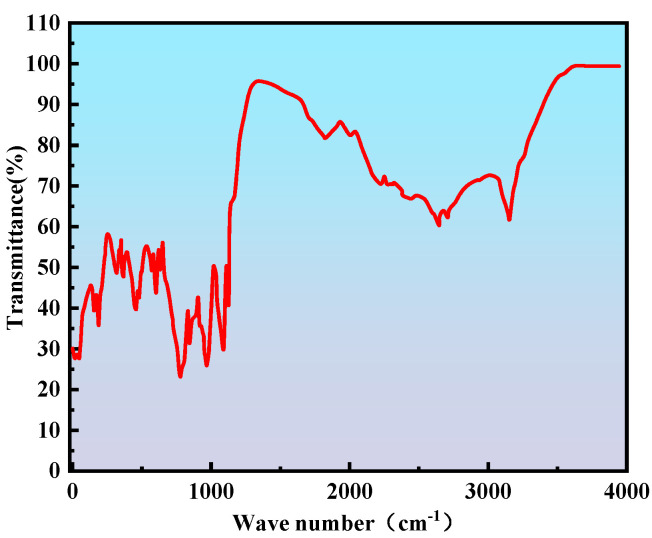
Infrared-spectrogram.

As shown in Figure 5 below. Fourier transform infrared spectroscopy (FTIR) characterization revealed distinct absorption peaks in the critical wavenumber regions of the gel system, reflecting the underlying molecular interactions. A strong absorption band at 1159 cm^−1^ corresponds to the C–O stretching vibration, originating from unreacted hydroxymethyl groups (–CH_2_OH) on the aromatic rings of SMP and the newly formed ether linkages (–CH_2_–O–CH_2_–) generated via self-crosslinking of the phenolic resin. The band at 1182 cm^−1^, assigned to the symmetric stretching of C–N bonds in secondary amine structures (–NH–CH_2_–), confirms the occurrence of a Mannich condensation reaction between SMP hydroxymethyl groups and the primary amines of PEI, thereby establishing covalent crosslinking. The 1395–1409 cm^−1^ range corresponds to C–N stretching vibrations, with peak broadening indicating the formation of multi-level amine structures and highlighting the complexity of the crosslinked network. An absorption band at 1435 cm^−1^, attributed to C–H bending vibrations, increases in intensity with higher SMP content, suggesting that hydrophobic aromatic structures participate in stabilizing the three-dimensional network. A sharp peak at 1559 cm^−1^ is primarily ascribed to the asymmetric stretching of C=O groups in carboxylate structures (–COO^−^/–NH_3_^+^ ion-pair complexes), indicating complete ionization of carboxyl groups and their electrostatic association with protonated amines. The notable shift in this peak to lower wavenumbers relative to free carboxyl groups further confirms the formation of ionic crosslinking. Specifically, the carboxyl groups (–COOH) of APAM are fully ionized to –COO^−^ in simulated formation water and subsequently interact with protonated amines (–NH_3_^+^) of PEI to form stable ion-pair complexes. Additional peaks include the saturated C–H stretching vibration at 2918 cm^−1^, associated with methylene groups (–CH_2_–) along the APAM backbone and ortho-methyl substituents on SMP aromatic rings, and a broad N–H stretching band around 3300 cm^−1^. The latter’s breadth reflects the coexistence of protonated primary (–NH_2_) and secondary (–NH–) amines within an extensive intermolecular hydrogen-bonding network. Overall, the spectral features align closely with the proposed triple-synergistic mechanism and molecular design strategy, providing molecular-level evidence for the gel system’s structural robustness and functional adaptability in high-temperature, high-salinity reservoir environments.

#### 2.2.3. Thermogravimetric Analysis

**Figure 6:**
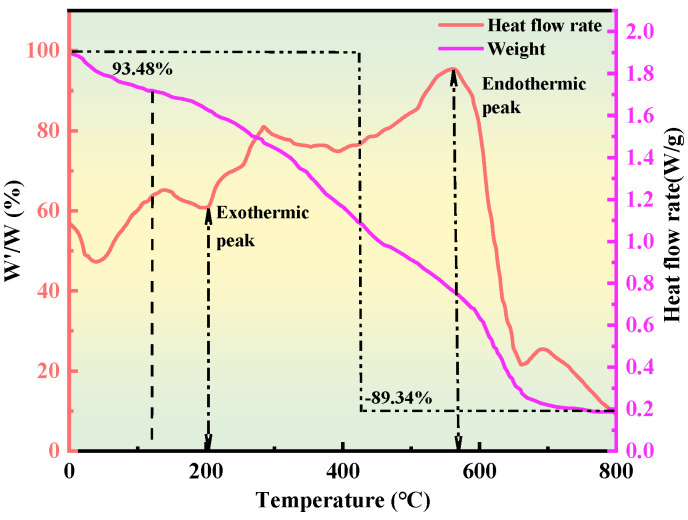
Thermogram-diagram.

Thermogravimetric–differential scanning calorimetry (TG–DSC) was employed over the temperature range of 35 °C to 800 °C to investigate the pyrolysis behavior of the gel system, examining changes in intermolecular interactions at different decomposition stages. This analysis also enabled the determination of the critical temperatures at which the gel network structure is disrupted, as well as the associated energy consumption during these processes. The measurements were performed on aged samples, and the resulting TG–DSC curves are presented in Figure 6.

Thermogravimetric analysis revealed that when the test temperature reached 120 °C, the gel system retained 93.48% of its initial mass, indicating that the gel used in this study is suitable for water-plugging applications under reservoir conditions up to 110 °C. Within this temperature range, minor weight loss was primarily attributed to the release of surface-bound water rather than scission of the polymer backbone. At this stage, only a fraction of the hydrophilic groups underwent thermal evaporation, while the majority of the molecular chains remained intact, without significant degradation. Upon further heating to approximately 200 °C, the gel underwent pronounced thermal decomposition, with pyrolytic depolymerization accelerating until around 500 °C, after which the degradation rate slowed. At this point, the principal hydrophilic functional groups in the gel structure—such as amide and carboxyl C=O bonds—were almost completely eliminated due to the sustained breakdown of the polymer backbone, leading to substantial thermal mass loss. Overall, the final mass loss over the entire temperature range reached 89.34%.

Differential scanning calorimetry further indicated that the gel system entered its glass transition at a relatively low temperature of approximately 82 °C, transitioning into a high-elastic state. As the temperature increased to around 190 °C, the gel began to cure, accompanied by an exothermic response, during which the rate of mass loss shifted from gradual to more rapid. Heating to approximately 200 °C initiated the melting stage, characterized by the scission of hydrophilic side chains and intensified dehydration. Upon reaching about 500 °C, the gel entered a decomposition–gasification phase, during which the progressive cleavage of the polymer backbone began to decelerate. By this stage, the gel had undergone substantial thermal dehydration and pyrolysis, demonstrating a favorable thermal resistance profile.

#### 2.2.4. Molecular Weight Analysis

**Figure 7:**
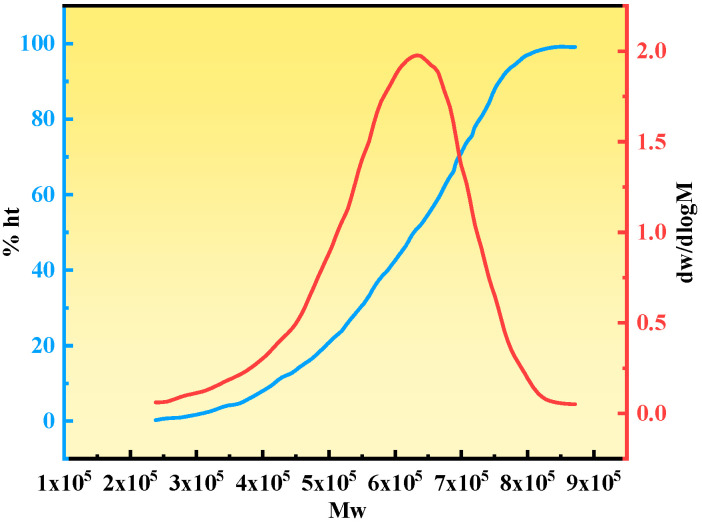
Molecular weight curve.

As shown in Figure 7 below. Molecular weight characterization revealed that the gel system exhibited excellent molecular weight uniformity, with a number-average molecular weight (Mn) of 135,050 g/mol, a weight-average molecular weight (Mw) of 173,435 g/mol, and a dispersity (Đ) of 1.284—significantly lower than the typical value for free-radical polymerization (Đ > 2.0). These results indicate several key features of the reaction system: (i) The PEI crosslinker precisely regulated the chain growth of APAM, effectively suppressing chain transfer side reactions; (ii) the dehydration–condensation rates of SMP hydroxymethyl groups and PEI amino groups were well-matched, leading to uniformly distributed high-molecular-weight polymers; (iii) the system overcame the conventional trade-off between gel strength and stability; and (iv) a quantitative predictive model was established linking molecular weight distribution to plugging performance. This provides an ideal plugging material for high-temperature and high-salinity reservoirs, combining high mechanical strength, slow dehydration, and rapid performance recovery.

#### 2.2.5. Rheological Curve Analysis

**Figure 8:**
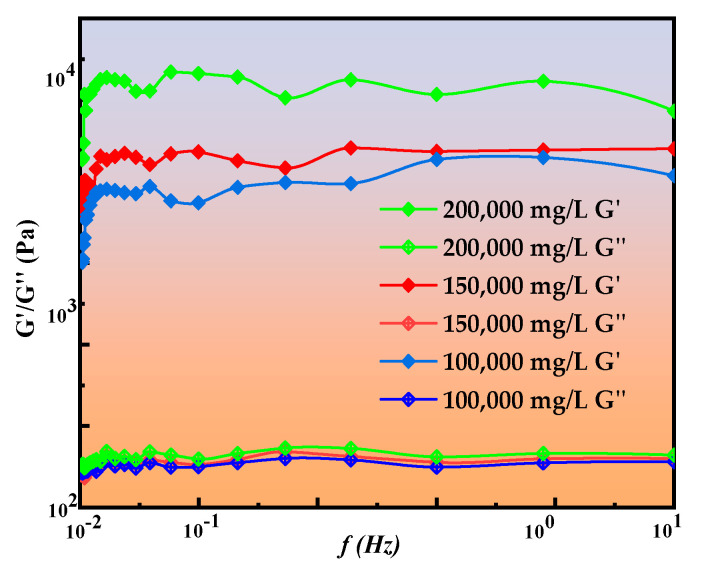
Gel-rheology.

Gel systems with superior recovery force and impact resistance after deformation exhibit stronger self-healing and re-crosslinking capabilities following local damage. To evaluate these properties, a HAAKE RS600 rheometer was employed to perform frequency sweep tests on the gel system across different frequency ranges, measuring its storage modulus (G′) and loss modulus (G″). The tests were conducted within a frequency range of 0.01–10 Hz under a shear stress of 0.2 Pa. The corresponding scanning results are presented in Figure 8.

The rheological properties of the gel system exhibited distinct characteristics under variations in shear frequency and temperature. Based on the data in Figure 8 (G′ = 4173 ± 95 Pa), three brine solutions with mineralization levels of 100,000 mg/L, 150,000 mg/L, and 200,000 mg/L were selected for testing within a frequency range of 0.01–10 Hz under a shear stress of 0.2 Pa. In all cases, the storage modulus (G′) and loss modulus (G″) increased slightly with shear frequency, indicating stable mechanical performance of the gel across different strain rates. The overall smooth rheological curves suggest enhanced crosslinking density within the gel network and strengthened intermolecular interactions. In the temperature range of 100 °C to 120 °C, G′ consistently exceeded G″, characteristic of elasticity-dominated viscoelastic behavior, confirming that the measured data fell within the viscoelastic regime. The gel existed as a viscoelastic solid, whose elasticity contributed to stable sealing of fractures and pore throats. With increasing temperature, G′ decreased, primarily due to intensified molecular thermal motion that weakened crosslinking or repulsive interactions, leading to reduced rigidity and diminished elastic recovery; at elevated temperatures, such changes could cause structural loosening. Under conditions of 110 °C, pH = 1–2, and a mineralization level of 150,000 mg/L, the gel exhibited G′ values in the range of 1543–4173 Pa, maintaining viscoelasticity, with elasticity as the dominant component. Gels prepared with simulated brine solutions were only slightly affected by temperature; however, mineralization had a pronounced effect on viscosity and strength before and after gelation. Possible reasons include (i) ionic interactions with polar groups of the polymer that weaken interchain attractions, (ii) ionic shielding effects forming a coating layer that hinders interchain interactions, and (iii) ion solvation effects that reduce intermolecular friction, thereby decreasing viscosity.

#### 2.2.6. Nuclear Magnetic Resonance Curve Analysis

**Figure 9:**
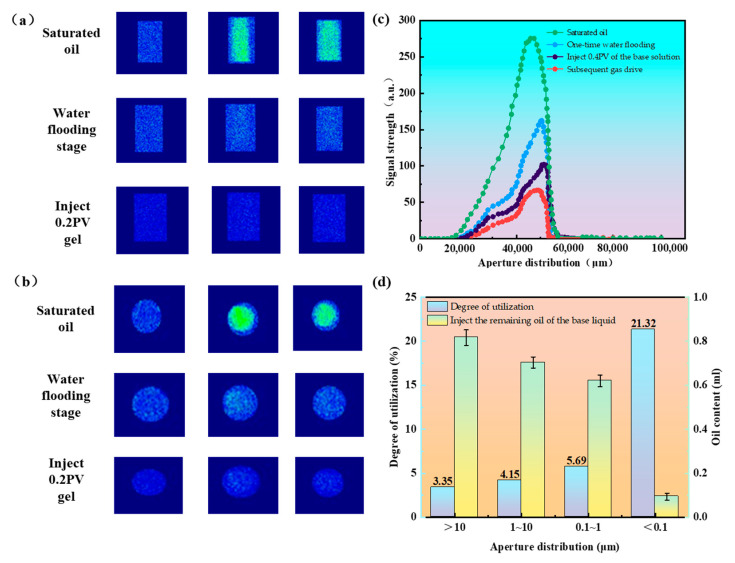
NMR imaging, T_2_ map, and pore activation map. (**a**) Coronal plane imaging comparison before and after injection into the gel system. (**b**) Cross-sectional imaging comparison before and after injection into the gel system. (**c**) T_2_ spectra of 50 mD core displacement at each stage. (**d**) The oil content distribution of the 50 mD core and the utilization degree of each pore diameter.

Throughout the displacement process, both water flooding and gel injection were introduced from the right end of the core, with the displaced fluids produced from the left end. According to the chromatic scale, the initial oil saturation is represented by color coding, where regions with higher oil content appear green, and those with lower oil content appear blue, as shown in Figure 9a,b. The images reveal that oil saturation within the core gradually increased, with distinct color changes observed across the three displacement stages. Ultimately, the remaining oil was predominantly confined to fine pore spaces, distributed relatively uniformly, with no evident zones of residual oil enrichment.

Following the completion of primary water flooding, 0.2 PV of the responsive gel precursor solution was injected. Upon completion, the nuclear magnetic resonance (NMR) T_2_ spectrum of the core was measured. As gel injection proceeded, the signal amplitude of the T_2_ spectrum further decreased. The oil saturation distribution, pore-size utilization, and contribution ratio of different pore sizes during gel injection are shown in Figure 9c,d. It can be observed that the pore structure exerted a significant influence on oil release during gel-based displacement. After the primary water flooding stage, the core retained a total of 2.20 mL of crude oil. Following gel precursor injection, the residual oil was mainly distributed across pores of various sizes: 0.82 mL in pores larger than 10 µm, 0.68 mL in 1–10 µm pores, 0.61 mL in 0.1–1 µm pores, and 0.05 mL in micropores smaller than 0.1 µm, which remained unrecovered during the primary water flooding. These results illustrate the spatial distribution of residual oil within the core and the contribution of different pore-size classes to oil displacement. During gel injection to 0.2 PV, the degree of pore utilization varied across size classes: approximately 3.35% for >10 µm pores, 4.15% for 1–10 µm pores, 5.69% for 0.1–1 µm pores, and 21.32% for micropores (<0.1 µm), representing the first mobilization of oil in the micropore fraction. The gel injection stage primarily mobilized oil from the 1–10 µm and >10 µm pore classes, while its effect on small and micropores was limited. By the end of the gel-based flooding stage, the incremental oil recovery was 2.63%, with residual oil predominantly remaining in >10 µm pores (19.63%) and 1–10 µm pores (17.11%), followed by 0.1–1 µm pores (14.35%), and only 0.97% in micropores (<0.1 µm).

### 2.3. Analysis of Core Oil Displacement Experimental Curves

#### 2.3.1. Microscopic Effects

**Figure 10:**
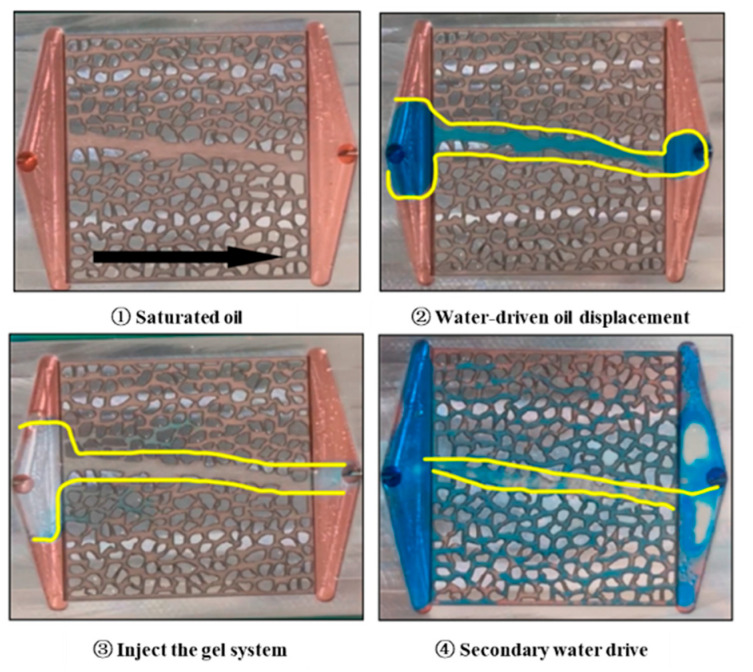
Microscopic visualization model of gel pore throat mobilization area (The yellow area represents the main working range of the gel).

After saturating the micro-visualization model with oil and aging it in an oven at 130 °C for 12 h, the subsequent displacement experiments revealed the complexity of multi-stage fluid interactions and their influence on displacement performance. The overall injection process is illustrated in Figure 10. In stage ①, the entire micro-model was saturated with oil. In stage ②, the water-phase flooding commenced. As the water phase primarily flowed along the fracture pathways, it advanced rapidly through the larger channels. However, this preferential flow behavior soon led to pronounced channeling within the fractures. Due to the relatively large flow conduits in the fractures, the water phase moved swiftly through these zones, bypassing the smaller pore throat regions adjacent to the fractures. As a result, the simulated oil in these regions was not effectively contacted and could not be efficiently displaced.

In stage ③, the gel base fluid was injected, revealing its potential advantages in fluid displacement. The introduction of the gel base fluid significantly alleviated the flow heterogeneity observed during the water flooding in stage ②. The gel base fluid was able to penetrate the regions adjacent to the fractures and progressively extend into the small pore throat areas on both sides of the fractures, particularly near the injection inlet. This process demonstrated that the gel base fluid could, to some extent, improve the distribution of fluids within the complex pore structure, enabling the mobilization of simulated oil in the small pore throat regions flanking the fractures. Nevertheless, the flow of the gel base fluid remained influenced by the fracture-dominated pathways; although its mobilization of oil in small pore throat regions was enhanced, the overall displacement effect still did not reach a fully uniform state.

Following an 8 h aging period of the gel base fluid, stage ④ was conducted as a secondary water flooding. Owing to the flow regulation achieved by the gel system, the secondary water flooding effectively controlled the swept volume and mitigated the channeling phenomenon observed during the primary water flooding in stage ②. As a result, simulated oil in the small pore throat regions on both sides of the fractures was further mobilized. In particular, the lighter color in these regions indicated a significant reduction in oil saturation.

#### 2.3.2. Parallel Experiment of Two Tubes

**Figure 11:**
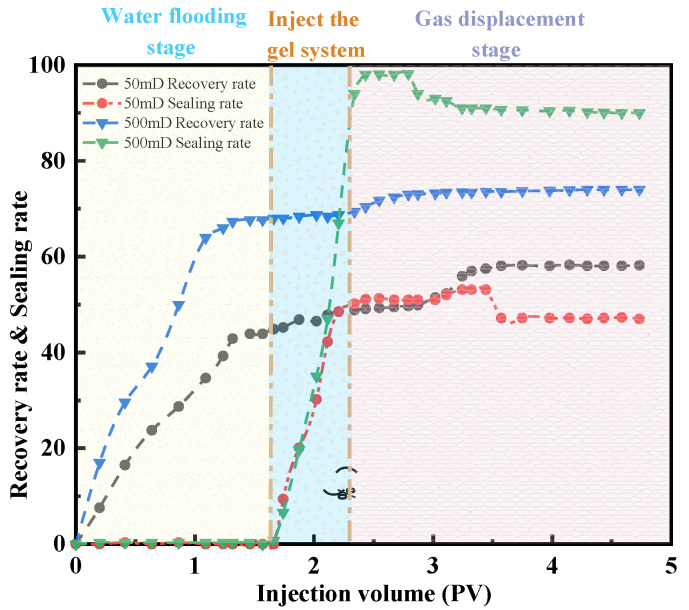
Block rate and recovery factor of parallel core.

To evaluate the plugging efficiency and oil recovery of the gel under different permeability conditions, parallel double-core flooding experiments were conducted. The results, as shown in Figure 11, illustrate the plugging efficiency and recovery factor for a low-permeability core (50 mD) and a high-permeability core (500 mD) at various injection volumes. After correcting the inlet pressure, it was observed that the gel system significantly increased the plugging resistance in the low-permeability core, thereby forcing the displacement fluid to preferentially flow toward the high-resistance zones under elevated flow resistance conditions.

For the low-permeability core, the oil recovery during the water flooding stage was 38.2%, which increased by 13.6% to reach 51.8% after gel injection. In contrast, for the high-permeability core, the recovery improved from 52.3% to 58.5%. The plugging performance of the gel system was further reflected in the increase in the plugging pressure in the low-permeability core from 0.18 MPa to 0.25 MPa, corresponding to a plugging efficiency of 38.9%. In the high-permeability core, the plugging pressure increased drastically from 0.05 MPa to 3.8 MPa, yielding a plugging efficiency of 98.7%. This indicates that the formation of a dense crosslinked network with a rigid structure created an immobile barrier, reducing the permeability from 500 mD to 6.5 mD. In the low-permeability core, only minor damage occurred, with permeability decreasing from 50 mD to 30.5 mD. From a recovery standpoint, these results elucidate the gel’s displacement mechanism: The effective plugging of the high-permeability layer forced the displacement fluid to be redirected toward the low-permeability zone, leading to a pronounced recovery enhancement. The gel formed an immobile barrier in the high-permeability layer (plugging efficiency >98%) while maintaining mobility in the low-permeability layer. Under 120 °C conditions, the gel exhibited a strength retention of over 90% after 30 days and a dehydration rate of less than 3%. The experiments confirm that under a permeability contrast of 10:1, the gel system achieved efficient plugging in the high-permeability layer and effective displacement in the low-permeability layer. The redistribution of flow paths was identified as the fundamental reason for the 13.6% increase in recovery in the low-permeability core.

## 3. Conclusions

(1) The study optimized the gel system’s synthesis parameters using response surface methodology (APAM 180 mg/L, PEI:SMP = 3:1, salinity 150,000 ppm, 110 °C) and established the relationship between APAM concentration and system viscosity over time. Based on this, quantitative analysis can be used to adjust the crosslinker concentration, thus controlling the progress of the crosslinking reaction. This approach enables more efficient planning of field oil well injection and shut-in waiting time for gelation, ensuring precise sealing of fractures in different reservoir sections. The gel system demonstrated a narrow molecular weight distribution (M_n_ = 135,050 g/mol, M_v_ = 173,435 g/mol, Đ = 1.284), significantly outperforming conventional free-radical polymerization systems (Đ > 2.0). This narrow dispersity promotes network uniformity and self-healing ability. The uniform molecular weight distribution ensures the nanoscale regularity of the crosslinked network, enabling the gel to exhibit rapid self-healing under shear stress (recovery rate > 98%). This study proposes a new paradigm for controlling network topology through molecular weight distribution (with plugging efficiency deviation <3% when Đ < 1.3) and establishes a quantitative predictive model for optimizing performance.

(2) The gel system exhibits excellent thermal stability, with a mass retention rate of 93.48% ± 1.02% at 120 °C, making it suitable for reservoir environments at 110 °C. Significant decomposition begins at 200 °C, and mass loss reaches 89.34% at 500 °C. Rheological performance shows that the gel predominantly behaves as a viscoelastic solid in the range of 100–120 °C, with the storage modulus (G′) exceeding the loss modulus (G″). At 110 °C, G′ ranges from 1543 to 4173 Pa, demonstrating strong resistance to erosion and excellent self-healing ability. Through rational formulation and structural optimization, the gel system performs exceptionally in terms of thermal and salt resistance, sealing capability, and oil displacement efficiency, offering an effective solution for the development of high-temperature, high-salinity reservoirs.

(3) Nuclear magnetic resonance imaging, micro-visualization, and parallel core displacement experiments demonstrate that the gel regulates fluid path distribution, reducing the residual oil saturation in large pores (>10 μm) to 19.63%. The underlying mechanism reveals that the gel preferentially enters high-permeability channels, forming an immobile barrier that forces subsequent displacing fluids to divert toward the low-permeability regions. This process addresses the inherent challenges of bypass flow and inefficient circulation in heterogeneous reservoirs. Through a comprehensive innovation chain involving molecular design, structural regulation, and performance validation, the gel system’s triple-synergistic crosslinking mechanism, narrow molecular weight distribution network, and selective sealing ability provide technical support for the development of deep heterogeneous reservoirs, offering both engineering applicability and environmental friendliness.

## 4. Materials and Methods

### 4.1. Materials and Instruments

Anionic polyacrylamide (APAM), high-salt-resistance crosslinking agent (polyethyleneimine PEI), high-temperature stabilizer (Phenolic resin SMP), sodium chloride (NaCl), magnesium chloride (MgCl_2_), calcium chloride (CaCl_2_), Aladdin Biochemical Technology Co., LTD.

NDJ-9S digital viscometer, Shanghai Lichen Bangxi Instrument Technology Co., LTD. Quanta 200F field emission scanning electron microscope, FEI Company, USA EVOMA15/LS15 scanning electron microscope, German Zeiss Company. Type 101-1 Electric Heating Constant Temperature Drying Oven, Gongyi Yuhua Instrument Co., LTD. HT-PNMR12-9 nuclear magnetic resonance spectrometer, Haihuantong Science and Education Equipment Co., LTD. FT-IR Fourier transform infrared spectrometer, Bruker, Germany TGA2(LF). Thermogravimetric analyzer, Mettler Toledo Technology (China) Co., LTD. GelMax-2000 gel chromatograph, Shanghai Du Kai Biotechnology Co., LTD. HAAKE rheometer (RS600), Thermo Fisher Scientific, Germany. Hx-ii high-temperature and high-pressure displacement oven, Nantong Huaxing Petroleum Instrument Co., LTD.

### 4.2. Gel Preparation Process

In a beaker, 100 mL of distilled water was added to prepare a high-salinity solution, which was then placed on a magnetic stirrer platform to begin stirring. A specific mass fraction of APAM was weighed and slowly added to the solution until all solids were completely dissolved, forming a uniform transparent solution. Next, high-salt-tolerant crosslinker (PEI) and high-temperature stabilizer (SMP) were gradually added until the total monomer concentration reached the designed value. The prepared gel solution was transferred into a sealed test tube and placed in a high-temperature oven. Viscosity was measured every 4 h. Crosslinking was considered to have begun when the viscosity exceeded 1000 mPa·s and continued to rise rapidly, until the gel strength reached level I or higher. Each sample was tested only once, and the sample was discarded once opened.

### 4.3. Optimize the Optimal Reaction Conditions

#### 4.3.1. The Response Surface Method Is Used to Analyze the Interaction Between the Two Factors

To optimize the gel system’s strength, the Box–Behnken response surface design method was employed as an effective strategy [26]. Based on the extensive results from previous single-factor experiments, it was determined that APAM concentration, PEI concentration, salinity, and temperature have significant effects on the synthesis outcome. The optimal ranges for these factors were established: APAM concentration between 150 mg/L and 210 mg/L, PEI:SMP concentration ratio between 200 mg/L and 400 mg/L, salinity between 100,000 ppm and 200,000 ppm, and temperature between 100 °C and 120 °C. To further optimize the parameters and identify the best formulation, a 4-factor, 3-level experiment was designed to evaluate the combined effect of these 4 factors on gel strength. Each factor was set at three levels: low (−1), medium (0), and high (1). By combining the different levels of these factors, the influence of each factor on gel strength could be clarified without the need for a full-factorial experiment. The experimental goal was to measure the gel strength score, with a range of 1 to 100, where higher scores indicate better gel strength. The optimal formulation conditions were determined, and the experimental factors and levels are shown in Table 2.

#### 4.3.2. Time–Viscosity Relationship

Based on the results from Section 2.1.1, a numerical fitting approach was employed to establish the relationship between the two most significant interacting factors, aimed at simulating the precise profile adjustment in practical applications [13]. The data obtained in Section 2.1.1 were subjected to Boltzmann fitting, as described by Equation (2).

(2) $$\eta \left(t\right)=\eta_{0}+\frac{\eta_{max}-\eta_{0}}{1+e^{\left(t_{0}-t\right)/{∆}_{t}}}$$

In the equation, η represents viscosity (mPa·s), $\eta_{0}$ is the initial viscosity (mPa·s), $\eta_{max}$ is the maximum viscosity (mPa·s), *t* is time (h), $t_{0}$ is the time corresponding to the curve inflection point (h), and ${∆}_{t}$ is the time difference for viscosity change (h). The system viscosity (η) follows an exponential relationship with time (*t*), and the viscosity variation curves under different independent variable factors exhibit similar patterns. This behavior can be summarized by the time–viscosity relationship in Equation (2).

### 4.4. Evaluation and Analysis of Gel System

#### 4.4.1. SEM Electron Microscope

Scanning electron microscopy (SEM) imaging provides direct visualization with high magnification, making it ideal for characterizing the microstructure of test samples. The sample to be tested was placed in a Petri dish and subjected to liquid nitrogen freezing to preserve its microstructure [27]. The frozen sample was then transferred to a freeze-drying instrument and vacuum-dried for approximately 8–10 h to remove moisture. Following this, the sample underwent ethanol gradient dehydration (30–100%, each stage lasting 15–30 min) and was then dried using a critical point dryer. In this process, liquid CO_2_ was used to replace the solvent in the sample (3–5 exchanges), and drying was completed under critical point conditions (31.1 °C/72.8 atm) to prevent surface tension-induced damage. After treatment, the sample was securely mounted onto the SEM copper sample holder using conductive adhesive. For SEM observation, a 3 kV accelerating voltage and a 5 mm working distance were selected, with secondary electron detection to capture surface morphology, facilitating detailed observation.

#### 4.4.2. Infrared Spectrum

Fourier transform infrared spectroscopy (FTIR) is based on the selective absorption of infrared light at specific wavelengths by molecular structures. The gel system to be tested was freeze-dried, and a 2 mg sample was mixed with dried potassium bromide powder (approximately 1:100 mass ratio). The mixture was then ground evenly and pressed into a transparent thin film. This film was directly coated onto a potassium bromide crystal to form a uniform liquid membrane. The FTIR scan range was 4000–400 cm^−1^, with a resolution of 0.35 cm^−1^ and 32 scans. The sample was placed in the optical path for scanning. After obtaining the spectrum, characteristic absorption peaks (e.g., C=O stretching vibrations, C-F bonds) were identified and compared with a standard spectral library to confirm the functional group structure.

#### 4.4.3. Thermogravimetric Analysis Method and Principle

A 10 mg sample was placed in an alumina crucible and treated in a vacuum-drying oven at 40 °C for 12 h. The crucible was then placed in a thermogravimetric analyzer, with a nitrogen atmosphere set at a flow rate of 50 mL/min. The temperature range was 25–800 °C, with a heating rate of 10 °C/min. A blank crucible was run simultaneously to account for baseline drift. The weight loss curve was recorded [28], and the initial decomposition temperature (*T*_0_), the temperature of maximum weight loss rate (*T_max_*), and the residual carbon content were analyzed to assess thermal stability and decomposition behavior. The gel system’s weight retention (*W′/W*) was evaluated. Subsequently, the glass transition temperature (*Tg*), crystallization temperature (*Tc*), and melting temperature (*Tm*) of the gel system were determined. The mass change (*W/g*) of the gel sample was observed under controlled temperature conditions, and differential scanning calorimetry (DSC) results were analyzed using the Gordon–Taylor equation.

(3) $$\sum_{i=0}^{n}W_{i}\cdot A_{i}\cdot \left(T_{g}-T_{g,i}\right)=0$$

In the formula, $W_{i}$ is the weight fraction of unit i, $T_{g}$ is the glass transition temperature, *A_i_* is the constant, and $T_{g,i}$ is the glass transition temperature of the homopolymer of unit i.

#### 4.4.4. Molecular Weight Analysis Method and Principle

The test sample was dissolved in a mobile phase of DMF containing LiBr at a concentration of approximately 5 mg/mL. The solution was subjected to ultrasonic treatment for 30 min until fully dissolved. It was then filtered through a 0.22 µm PTFE membrane to remove insoluble substances and particulate impurities. The gel chromatography system was activated, and a suitable chromatographic column was selected. The mobile phase flow rate was set to 1.0 mL/min, and the column temperature was maintained at 30 °C. A calibration curve was established using narrow-distribution polystyrene standards, covering the target molecular weight range (1k–500k Da). A 20 µL sample was injected, and the elution curve was recorded using a differential refractive index detector (RID). The number-average molecular weight (*M_n_*), weight-average molecular weight (*M_v_*), and dispersity index (*Đ*) were calculated [29].

#### 4.4.5. Rheological Experiment

Gel-based water shutoff systems exhibit a non-Newtonian fluid behavior, positioned between elastic solids and viscous liquids. Rheological properties, especially viscoelasticity, are commonly used to characterize gel systems. The mechanical properties of the gel structure change over time as it relaxes. The storage modulus (*G′*) reflects the elastic nature of the gel, while the loss modulus (*G″*) indicates its viscosity [30]. A higher loss modulus (*G″*) implies greater internal friction and resistance to erosion, whereas a higher storage modulus (*G′*) signifies better recovery and impact resistance after deformation. The gel’s self-healing and re-crosslinking ability are stronger after localized damage. Under mechanical deformation, part of the energy appears elastically, corresponding to the stress response described by the storage modulus (*G′*), while another portion of energy is dissipated based on the molecular structure during dynamic movement, reflected by the stress response of the loss modulus (*G″*). During the testing process, it was confirmed that the storage modulus (*G′*) was higher than the loss modulus (*G″*), ensuring that all dynamic rheological tests were conducted within the stable viscoelastic region. The storage modulus (*G′*) and loss modulus (*G″*) of the gel system before and after gelation were measured using a HAAKE rheometer for viscoelastic evaluation, with a shear rate of 7.34 s^−1^. The relationship between *G′*, *G″*, and the loss angle δ is given by the following equation:

(4) $$\tan\delta =G^{\prime \prime }/G^{\prime }$$

In the formula, δ represents the fluid property, δ = 0–20° indicates an elastomer, δ = 20–40° indicates a viscoelastomer, and δ > 40° indicates a viscous substance.

#### 4.4.6. Magnetic Resonance Testing

**Figure 12:**
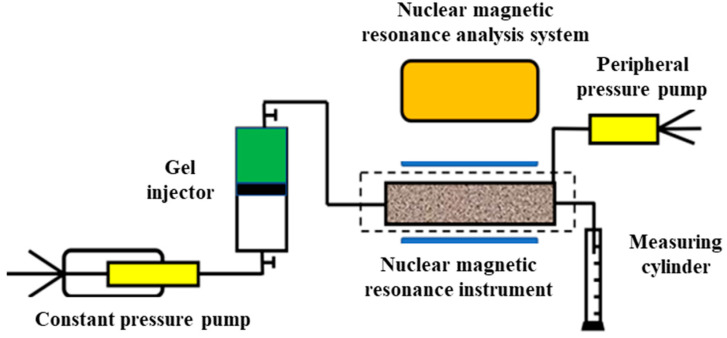
NMR system.

The nuclear magnetic resonance (NMR) T_2_ spectrum is used to describe the pore distribution characteristics of rock cores by analyzing the distribution of fluids within the porous medium. The relaxation time reflects only the pore size but does not fully describe the pore diameter of the porous medium. Mercury intrusion porosimetry, which calculates pore size based on capillary pressure, provides a more detailed measurement. By combining NMR technology with mercury intrusion porosimetry, a permeability of 25.8 mD was selected. The NMR T_2_ spectrum was compared with the pore throat distribution obtained from the mercury intrusion curve. In addition, thin-section data of the core were considered to establish a correlation between relaxation time and pore diameter [31]. The NMR testing system used in the experiment is shown in Figure 12 below.

The *T*_2_ spectrum in porous media is expressed according to the following formula:

(5) $$T_{2}=\frac{1}{T_{2S}}+\frac{1}{T_{2D}}+\frac{1}{T_{2B}}$$

In the formula, *T*_2*S*_ is the surface relaxation term, ms; *T*_2*D*_ is the diffusion relaxation term, ms; and *T*_2*B*_ is the body relaxation term, ms. Nuclear magnetic resonance technology is used to determine the fluid in porous media. The diffusion relaxation and bulk relaxation terms can be ignored, and the relaxation time of the fluid mainly depends on the surface relaxation term. The surface relaxation term is related to the specific surface area of the rock: The relaxation term increases as the specific surface area of the rock increases, and the *T*_2_ relaxation time decreases. The surface relaxation term of the rock can be expressed by the following formula:

(6) $$\frac{1}{T_{2S}}=\rho_{2}\frac{S}{V}$$

In the formula, Ρ_2_ is the relaxation rate, μm/ms, and S/V is the specific surface area of the pore throat, 1/μm. The specific surface area is related to the pore throat radius of the porous medium:

(7) $$\frac{S}{V}=\frac{F_{S}}{r}$$

*F_S_* is the dimensionless shape coefficient of the pore throat, and *r* is the pore throat radius, μm. Therefore, *T*_2_ can be expressed as:

(8) $$T_{2}=\frac{1}{\rho_{2}F_{S}}r^{n}$$

The degree of micropore utilization (U) is used to quantitatively characterize the proportion of the gel that actually occupies the target pore structure:

(9) $$U=\frac{V_{used}}{V_{total}}\times 100\%$$

where $V_{used}$ is the volume of micropores occupied by the gel after sealing (measured by low-field nuclear magnetic resonance, NMR), in cm^3^, and $V_{total}$ is the total micropore volume of the target area (calculated from the total core volume and porosity), in cm^3^.

### 4.5. Double-Tube Parallel Core Experiment

#### 4.5.1. Gel-Sealing Mechanism and Microscopic Visualization Experiment

The mechanical initiation mechanism of the composite gel water-blocking system simulated by indoor experimental physics follows the Navier–Stokes equation of fluid dynamic motion:

(10) $$\frac{\partial \alpha \rho }{\partial t}+\nabla \cdot \rho \alpha \nu =0$$

In the formula, *α* is the fluid volume fraction; *ρ* is the fluid density, kg/m^3^; and *ν* is the fluid velocity, m/s.

Due to the continuous accumulation of the continuous-phase gel during the migration process, the interaction with the fluid generates action and reaction forces. According to Newton’s second law, the forces acting on the gel system mainly include gravity, the interaction force between the polymer and the crosslinking agent, and the interaction force between the gel system and the fluid. Among them, drag forces will be generated between the two mutually moving phases. Based on this, the motion equation of the gel system can be expressed by Formula (14):

(11) $$m\frac{d\nu_{p}}{dt}=F_{D}+F_{g}+\sum F_{i}$$

In the formula, $\nu_{p}$ is the gel migration velocity, m/s; $F_{D}$ is the fluid drag force, N; $F_{g}$ is gravity, N; and $F_{i}$ is the the force between different gel systems, N.

The gel system follows the equation of conservation of momentum during fluid motion:

(12) $$\frac{\partial \alpha \rho }{\partial t}+\nabla \cdot \rho \alpha \mu \nu =-\nabla \rho +\nabla \cdot \left(\mu \alpha \nabla \nu \right)+\rho \alpha g-F_{D}$$

In the formula, *g* is the gravitational acceleration, m^2^/s; *μ* is the fluid viscosity, kg/(m·s); and *F_D_* is the fluid drag force, N.

**Figure 13:**
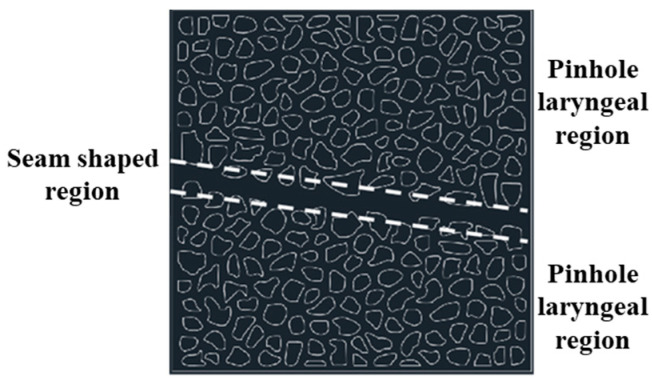
Microscopic model.

The creation of a microscopic model for a high-temperature and high-pressure visual microscopic displacement experiment involves the following steps: Design the model: Based on the experimental requirements, design the geometric shape and dimensions of the model. The commonly used model is made of transparent high-strength plastic material, allowing for a clear observation of fluid flow and displacement processes. Material selection: Use transparent materials resistant to high temperature and high pressure, such as special glass. It has good chemical corrosion resistance to adapt to the experimental environment. Mold making: Make the model mold according to the design drawings. The model shown in Figure 13 below is the model designed for the laboratory. The precision of the mold directly affects the quality of the final model, which is manufactured using 3D printing technology. Injection of material: Inject the selected transparent material into the mold to ensure uniform filling. For resin models, bubbles need to be removed in a vacuum environment after injection. Curing and processing: Subject the injected material to a curing treatment. After completion, remove the model from the mold. Carry out subsequent processing on the model and polish the surface. Install accessories: Install accessories on the model, including injection holes, sampling ports, and pressure sensor interfaces. Conduct experiments: After the model is completed, conduct high-temperature and high-pressure tests on it to verify the performance and stability of the model. Ensure that under experimental conditions, the model can work properly and provide clear visualization effects.

This model adopts an alternating configuration of small holes and slit structures, and the width of the slit structure is relatively narrow. This design is used to study the influence of alternating gaps and apertures on fluid behavior. Effective size: 30 × 30 mm. Small holes and narrow slits are alternately arranged, and the junctions of small holes and slits form specific fluid channel characteristics. Analyze the alternating effects of gaps and apertures on fluid flow and displacement behavior, as well as their manifestations under different structures.

#### 4.5.2. Parallel Displacement Experiment

One group each of low-permeability (50 mD) and high-permeability (500 mD) standard sandstone cores (with a diameter of 2.5 cm and a length of 10 cm) was selected. After cleaning and drying, the simulated formation water (with a salinity of 150,000 PPM) was vacuum-saturated and installed in the parallel core clamps. The confining pressure was maintained at 15 MPa, and the temperature was set as the reservoir temperature (60 °C). The simulated formation water was injected at a constant flow rate (0.1 mL/min), the pressure difference at the inlet of the two cores was recorded [32], and the initial permeability was calculated:

(13) $$K=\frac{Q\mu L}{A\cdot ∆P}$$

In the formula, K is the permeability, 10^−3^ $\mu m^{2}$; *Q* is the flow rate at the core outlet, mL/min; mu is the viscosity of simulated formation water, and is taken as 8.9 × 10^−4^ Pa·s; *L* is the core length, cm; *A* is the cross-sectional area of the core, cm^2^; and *∆P* is the pressure difference at both ends of the core, MPa.

The gel was prepared and pre-crosslinked for 30 min. The gel was injected into the two cores at a rate of 0.3 mL/min. The injection was stopped when the pressure at the outlet end of the hypertonic core rose to three times the initial pressure or the flow rate of the hypotonic core decreased by 90%. The cumulative injection volume was recorded. The system was shut down and let stand for 24 h (at a constant temperature of 60 °C) to ensure that the gel was fully cross-linked to form a three-dimensional network structure. The formation water was injected again at 0.1 mL/min to monitor the changes in the flow rate at the outlets of the two cores until the pressure stabilized. The post-blockage permeability *K* and the water-blocking rate Γ were calculated:

(14) $$\Gamma =\frac{K_{W}-K_{P}}{K_{W}}\times 100\%$$

In the formula, $K_{W}$ and $K_{P}$ are the water permeability measured before and after the injection of the gel blocking agent, respectively.

To avoid flow deviations caused by instrument system errors, a pre-pressurization test was conducted on each core channel under idle conditions before the experiment. This allowed for the calibration of the initial inlet pressure, ensuring that any subsequent pressure changes during the injection phase were attributed solely to the effects of the gel system.

## Figures and Tables

**Table 1:** Model ANOVA.

Source	Sum of Squares	Degree of Freedom	Mean Square	F-Value	*p*-Value
Model	1201.88	14	85.85	9.13	<0.0001
A	4.08	1	4.08	3.54	0.0793
B	33.33	1	33.33	0.4341	0.5200
C	0.7500	1	0.7500	0.797	0.7815
D	21.33	1	21.33	2.27	0.1528
AB	2.25	1	2.25	0.2392	0.6318
AC	1.00	1	1.00	0.1063	0.7489
AD	9.00	1	9.00	0.9569	0.3435
BC	1.00	1	1.00	0.1063	0.7489
BD	0.2500	1	0.2500	0.0266	0.8727
CD	0.2500	1	0.2500	0.0266	0.8727
A^2^	1015.05	1	1015.05	107.92	0.0023
B^2^	126.30	1	126.30	13.43	<0.0001
C^2^	165.76	1	165.76	17.62	0.0008
D^2^	86.01	1	86.01	9.14	0.0085
Residual	141.08	15	9.14	/	/
Items not drafted	131.08	10	13.11	6.55	0.0855
Pure error	0.1280	5	0.0520	/	/
Total sum	1332.97	29	/	/	/

**Table 2:** Variable factors and levels.

Level	Factor			
	A. APAM (mg/L)	B. PEI:SMP	C. TDS (ppm)	D. Temperature (°C)
−1	150	2:1	100,000	100
0	180	3:1	150,000	110
1	210	4:1	200,000	120

## Data Availability

The data presented in this study are openly available in the article.

## References

[1] Liu J., Zhong L., Wang C., Li S., Yuan X., Liu Y., Meng X., Zou J., Wang Q. (2020). Investigation of a high temperature gel system for application in saline oil and gas reservoirs for profile modification. J. Pet. Sci. Eng..

[2] Zhai Y., Fang Z., Feng J., Sun C., Deng W., Wen Y. (2024). Development and performance evaluation of re-crosslinkable preformed particle gel under high temperature and high salt conditions. Polymer.

[3] Wei Z., Liu J., Zhang L., Yang L., Li Y., Wang J., Fan H. (2025). A novel approach to investigating the influencing factors and mechanism of re-crosslinking performance of re-crosslinkable preformed particle gels. Geoenergy Sci. Eng..

[4] Peng Y., Ye J., Li Y., Chen Y., Li Z., Zhang D. (2025). Development and Performance Evaluation of Novel Self-Degradable Preformed Particulate Gels with High-Temperature and High-Salinity Resistance. SPE J..

[5] Xin H., Fang B., Yu L., Lu Y., Xu K., Li K. (2023). Rheological performance of high-temperature-resistant, salt-resistant fracturing fluid gel based on organic-zirconium-crosslinked HPAM. Gels.

[6] Xu B., Wang Y. (2021). Profile control performance and field application of preformed particle gel in low-permeability fractured reservoir. J. Pet. Explor. Prod. Technol..

[7] Deng J., Lian H., Zhuang Y., Zhao H., Wang Z., Tian Y., Lin C., Yuan H., Han M., Lu G. (2024). Synthesis and performance evaluation of multi-crosslinked preformed particle gels with ultra-high strength and high-temperature and high-salinity resistance for conformance control. Fuel.

[8] Guo H., Ge J., Xu Y., Lv Q., Li Z., Zhou D., Tao Z. (2022). Preparation and Mechanism of Stability for High-Temperature and High-Salinity Gels. SPE J..

[9] Ge J., Guo H., Zhang T., Zhou D., Xu Y., Lü Q. (2022). Development of temperature and salinity resistant phenolic gel and its performance regulation mechanism. Acta Pet. Sin..

[10] Zhou G., Zhang X., Yan W., Qiu Z. (2025). Synthesis, Characteristics, and Field Applications of High-Temperature and Salt-Resistant Polymer Gel Tackifier. Gels.

[11] Wang Z., Bai B., Zhou E., Pu J., Schuman T.P. (2019). Experimental Evaluation of Oxidizing Breakers for a Polyacrylamide-Based Re-Crosslinkable Preformed Particle Gel. Energy Fuels.

[12] Liu J., Zhong L., Cao Z., Hao T., Liu Y., Wu W. (2022). High-temperature performance and cross-linking mechanism of different types of gel systems in saline environment. J. Appl. Polym. Sci..

[13] Wu Y., Zhang H., Zhang L., Huang Y., Zhao M., Dai C. (2024). Development of Novel Delayed Swelling Polymer Gel Particles with Salt Resistance for Enhanced In-Depth Permeability Control. SPE J..

[14] Shu Z., Qi Y., Luo P. (2023). Research and performance evaluation of modified nano-silica gel plugging agent. J. Appl. Polym. Sci..

[15] Bai Y., Wu L., Luo P., Li D. (2022). Synthesis and evaluation of delayed anti-high temperature gel plugging agent. Front. Energy Res..

[16] Chen J., Qiu H., Djouonkep L.D.W., Lv J., Xie B. (2024). Preparation, Evaluation and Field Application of Thermally Induced Crosslinked Polymer Gel Leakage Plugging Agent. J. Polym. Environ..

[17] Fang J., Sun J., Feng X., Pan L., Bai Y., Yang J. (2025). Research and Development of a High-Temperature-Resistant, Gel-Breaking Chemical Gel Plugging Agent and Evaluation of Its Physicochemical Properties. Gels.

[18] Liu J., Fu H., Luo Z., Chen W., Liu F., Zhao M. (2023). Preparation and performance of pH-temperature responsive low-damage gel temporary plugging agent. Colloids Surf. A Physicochem. Eng. Asp..

[19] Fan H., Pu W., Du D., Xu M., Chen L., Xu L., Jin F. (2023). Experimental study on the plugging and profile control of preformed particle gels in high temperature and salinity reservoir. J. Appl. Polym. Sci..

[20] Huang B., Zhang W., Zhou Q., Fu C., He S. (2020). Preparation and Experimental Study of a Low-Initial-Viscosity Gel Plugging Agent. ACS Omega.

[21] Li Y.-K., Hou J.-R., Wu W.-P., Qu M., Liang T., Zhong W.-X., Wen Y.-C., Sun H.-T., Pan Y.-N. (2024). A novel profile modification HPF-Co gel satisfied with fractured low permeability reservoirs in high temperature and high salinity. Pet. Sci..

[22] Liu F., Yin D., Sun J., Luo X., Huang X. (2024). Preparation and Characterization of Temperature-Sensitive Gel Plugging Agent. Gels.

[23] Tian J., Liu J., Liu S., Yuan S., Cai W., Jiang H. (2023). A gel system with long-term high-temperature stability for deep profile modification of high-temperature oil and gas reservoirs. J. Appl. Polym. Sci..

[24] Li Z., Zhao G., Xiang C. (2022). Synthesis and Properties of a Gel Agent with a High Salt Resistance for Use in Weak-Gel-Type Water-Based Drilling Fluid. Arab. J. Sci. Eng..

[25] Zou H., Wang Y., Xu Y., Li J., Wu L., Su G., Yu X., Yang H. (2024). Synthesis and Performance Study of Self-Degradable Gel Plugging Agents Suitable for Medium- and Low-Temperature Reservoirs. ACS Omega.

[26] Wang H., Yang C., Zhang Y., Wang C. (2024). Preparation and Effect of CO_2_ Response Gel for Plugging Low-Permeability Reservoirs. Gels.

[27] Jia H., Ren Q. (2016). Evidence of the Gelation Acceleration Mechanism of HPAM Gel with Ammonium Salt at Ultralow Temperature by SEM Study. SPE Prod. Oper..

[28] Zhang W., Gao R., Sha X., Liu G., Wang X.-Y., Lin R.-S., Tong Y. (2025). Strength development and thermal stability analysis of carbonation-cured red mud-based cementitious materials. Constr. Build. Mater..

[29] Papp D., Carlström G., Nylander T., Sandahl M., Turner C. (2024). A Complementary Multitechnique Approach to Assess the Bias in Molecular Weight Determination of Lignin by Derivatization-Free Gel Permeation Chromatography. Anal. Chem..

[30] Liu M., Ge J., Wu Q., Ma A., Li J., Zhang G., Fan H., Jiang P., Pei H. (2024). Study on temperature-resistance and salt-tolerance aluminum gel and its rheological analysis. Colloid Polym. Sci..

[31] Deng Z., Liu M., Qin J., Sun H., Zhang H., Zhi K., Zhu D. (2022). Mechanism study of water control and oil recovery improvement by polymer gels based on nuclear magnetic resonance. J. Pet. Sci. Eng..

[32] Zhang L., Liu Y., Wang Z., Li H., Zhao Y., Pan Y., Liu Y., Yuan W., Hou J. (2024). Evaluation of Profile Control and Oil Displacement Effect of Starch Gel and Nano-MoS_2_ Combination System in High-Temperature Heterogeneous Reservoir. Gels.

